# Hospital and Institutional News

**Published:** 1913-01-18

**Authors:** 


### HOSPITAL AND INSTITUTIONAL NEWS.

### THE CRISIS IN THE SUNDAY FUND.

A special meeting of the Council of the Metropolitan Hospital Sunday Fund will take place very shortly to consider, among other matters, a resolution of which the Rev. W. Hardy Harwood and Sir Henry Burdett have given notice as follows: "That the laws of the constitution of the Hospital Sunday Fund be referred to a small special committee of the Council to bring up rules or by-laws regulating the procedure which shall in future govern the proceedings at the meetings of constituents, and that these by-laws, when approved by the Council, shall be laid before a special meeting of the constituents to be held at the earliest possible date."

### ALMONERS AND INSURED PATIENTS.

How will the Insurance Act affect the almoner? This question is engaging attention in view of the general belief—or should we say hope?—that the Insurance Act will diminish the number of out-patients, possibly, it is thought, by 25 per cent. The Secretary of the Hospital Almoners' Council, in a letter which we publish this week, draws from some recent remarks of the Treasurer of St. Thomas's Hospital the conclusion that St. Thomas's, at all events, is convinced that only through the agency of the almoner can insured persons be properly dealt with. In the meantime the Hospital Saturday Fund has referred to a special committee, without discussion, the Distribution Committee's special report on the almoner system and the changes to be expected from the Insurance Act. The subject is well worth investigating for two reasons. In the first place, it will be a relief to institutions generally if one of their existing officials can be found capable of dealing with insured persons, for there is always a not unnatural reluctance to create an entirely new office. In the second place, supposing the predicted reduction of the numbers of out-patients ensues, it will be a distinct gain to the almoner by relegating to other hands the not very satisfactory policing and restrictive sides of her work, and so setting her more free to "follow up" cases and to co-ordinate after-care societies, for it is idle to suppose that the need for these efforts ceased automatically on January 15. Then, too, the after-care of in-patients has always been the most urgent side of the reconstructive problem, and the numbers of these are certainly likely to be increased by the addition of those whom panel doctors have none of the old temptation to keep out the hospitals' reach. This is the class which needs most help precisely at the time when it is the almoner's duty to step in.

### THE MIDDLESBROUGH GUARDIANS' OPPORTUNITY.

The meeting of the Middlesbrough Guardians held last week, over which Mr. G. Wycherley presided, had before it certain opinions of Dr. Fuller, a Local Government Board inspector, with reference to the workhouse hospital. Dr. Fuller, it appears, considered that the institution was not so efficient as the size and importance of the Union required, and added to his criticism the recommendation that the accommodation of the medical officer should be improved, and that the nursing staff should be increased by five. The consideration of his suggestions was deferred. Local Government Board inspectors are not usually free with their opinions unless they have good grounds for expressing them. We hope, therefore, that matters so urgent as an understaffed infirmary and a badly housed medical officer, for such apparently are what the inspector's words imply, will be looked into without a moment's avoidable delay, if only for the honour of the Middlesbrough Board of Guardians.

### THE TREATMENT OF INSURED PERSONS.

We beg to thank the numerous institutional secretaries who have forwarded to us copies of the resolutions which have been passed by their committees of management with reference to the admission of insured persons. A copy of the notices to be placed in the various departments, and of the letters which are being sent out to local practitioners and to governors and subscribers, has generally accompanied these resolutions, and has enabled us, through the kind co-operation of the managers, to have a very useful sample batch of documents for reference and comparison. Apart from their intrinsic value, we take the spontaneous sending of these documents as an indication of that continued co-operation between institutional workers and their Journal which we asked for when thanking our readers for the splendid response given to a request for personal accounts of the Christmas festivities.

### THE DISEASES OF HISTORICAL PERSONAGES.

A somewhat fascinating study, which has lately been absorbing the energies of various medical men with antiquarian leanings, is that of diagnosing from contemporary accounts the diseases which have affected historical characters. Some little while ago discussion grew heated on the question whether Milton's blindness was caused by glaucoma or detachment of the retina; just lately several other eminent persons have been subjected to paragnosis, if the term can be allowed. Louis XIV. unquestionably died of senile gangrene of the leg. A writer in an American paper shows that his physician quite failed to appreciate the gravity of the case until it was too late, whereas the Court surgeon made the diagnosis at once. Since in those days the surgeon was an inferior order of practitioner to the physician, the Grand Monarque's chance of recovery was speedily frittered away. Such is the danger of exclusiveness. Napoleon is known to have died of cancer of the stomach; and it has been suggested that the fit of indigestion which is supposed to have cost him the Battle of Waterloo was one of the early symptoms of the growth, or of an antecedent ulcer. Professor Keith, however, has just published some researches which lead him to believe that Napoleon suffered from Malta fever, possibly contracted in Elba, possibly in St. Helena. He does not, however, deny that gastric cancer was also present at the end of his life. Dr. Arnold Chaplin has also published lately a critical summary of the course of Napoleon's illness, from which it appears that the ex-Emperor's own physicians (O'Meara and Antommarchi) diagnosed inflammation of the liver, while the British official medical officer (Arnott) said it was nothing but hypochondriasis! Then, again, another author has investigated the case of a certain Duchess of Orleans, who was at the time suspected to have been poisoned, but is now shown to have had symptoms much more suggestive of a perforated ulcer of the stomach or duodenum. It is an unfortunate fact that the public is not educated enough to prefer such researches to the Court scandals that are so popular.

### THE MACDONALD MEMORIAL AT LEICESTER.

The "Margaret Macdonald" Memorial Committee, which was appointed to raise and administer a fund to perpetuate in Leicester the memory of Mrs. Macdonald, has decided to make use of the sum of £969 odd, which has been received for the Memorial, to name the new ward to be built in connection with the Children's Hospital. Sir Edward Wood, who promised to make up the amount to £1,000, reports that the board of governors of the infirmary had given their hearty approval to the scheme. A commemorative stone, with the following inscription, will be placed in the new ward:—"This ward is dedicated to the memory of Margaret Macdonald, wife of J. Ramsay Macdonald, M.P., who loved little children and strove to bless them. 1913." Mr. C. J. Bond, F.R.C.S., has acted as chairman of the movement, and Mr. H. H. Peach has carried out the duties of hon. secretary.

### POETRY AND MEDICINE.

Three years ago, on December 25, 1909, we published an article dealing with this subject entitled "Francis Thompson, Poet and Medical Student." That article evidently has not been wasted. Its latest fruit appears to be to bring us the January number of the *Poetry Review*, of which Mr. Stephen Phillips has been appointed editor. In the article on Francis Thompson, which was an enthusiastic but careful appreciation of his verse and prose, the writer incidentally showed how the language of science lends itself to poetry quite as well as, for example, the language of agriculture, which has always been so popular with the poets. The *Poetry Review*, however, which we believe is the only Review of its class in this country devoted wholly to poetry, naturally takes a wider field, and is chiefly noteworthy for two reasons. First, it is offering a premium of £5 per month for the best poem of not more than 250 and not less than 40 lines. These premiums have been very rightly noised abroad in the newspapers, and doubtless have created much excitement among minor poets and poetesses, some of whom surely exist in the institutional world; but the very important condition was not previously announced—namely, that "all competitors must be members of the Poetry Society," to enter which costs half a sovereign. It seems rather hard that any acute ones who were fired by the announcements of the premiums some weeks ago and hurriedly sat up one night composing, or drew from a dusty drawer a previously written poem of the required length, should be penalised for their promptitude in sending in their manuscripts. Lord Dunsany's play, "The Gods of the Mountain," appears in this issue, and connoisseurs of imaginative literature will purchase it on that account. The influence of "The House of Pomegranates" is apparent in his style. It is related of Lord Dunsany that he described the word "caption" as "the word of shame, the word that may not be spoken." What does he think of "audition" which appears about a dozen times, and only once with the doubtful chaperon of inverted commas? Is a Review which can command his pen so poor in English that it must employ a word so hideous and of such doubtful propriety? Having often maintained that poetry and medicine impinged on the same spheres, and that the institution offered quite as fine an opportunity to the man of letters as the public school, which is the only establishment which so far has found a place in first-rate fiction, we make no apology for informing institutional workers of the opening for their literary talents which is offered by our contemporary.

### A NEW SCHOLARSHIP FOR WOMEN DOCTORS.

The London Royal Free Hospital School of Medicine for Women has received a bequest from the late Miss Mabel Sharman-Crawford to found a scholarship bearing her name. In value £20 for four years, it will be offered for the first time in July on the result of an examination in biology, chemistry, and physics. Particulars are to be obtained from the Secretary of the School at 8 Hunter Street, W.C. It is not too common for testators to endow or make attractive new branches of study, though no thoughtful authority would deny that to help on a study not at present generally well supported should be the aim of wise founders. There is a fashion in these, as in other matters, though the nature of this bequest would seem to show that Miss Sharman-Crawford had her own reasons for singling out the above three subjects. There are local needs as well as national ones, and we trust that this new foundation may bring further honours to the London School of Medicine for Women.

### THE VALUE OF THE OFFERTORY.

The customary analysis of the contributions made by the various religious bodies on Hospital Sunday in London, which appears annually in the *National Church*, is announced. The total subscribed was £35,840, being £1,200 odd less than the amount realised in 1911. We extract the first six denominations from the list, excluding shillings and pence from the totals: Church of England, £28,102;

Jews, £1,472; Congregationalists, £1,470; Presbyterians, £951; Wesleyans, £811; Roman Catholics, £680. The Church of England contributed rather more than three-quarters of the sum total, and the offertory of St. Paul's Cathedral heads the list with £4,214, rather more than the next three denominations subscribed collectively. Hospital Sunday in London is, of course, a different thing from Hospital Sunday in the provinces, from which, indeed, the Metropolis derived the idea, which is now in its fortieth year. The moral, however, to be drawn from these figures is that £36,000 is too large a sum to be made the sport of sectional interests, and that the solidarity which the voluntary system has done so much to create in institutional workers, and between the sick and the well, should be strong enough to check any institution from risking, in the personal hope of gain, the success of the fund in general by raising money in any form likely to offend the majority of the subscribers.

### THE LATE NATHANIEL COHEN.

A prominent member of the Jewish community, who characteristically interested himself in the voluntary side of institutional work, has left us by the death last Tuesday of Mr. Nathaniel Louis Cohen. After retiring from the banking firm, which was dissolved in 1901 by the mutual consent of the partners, Mr. Cohen took an interest in many sides of philanthropic work. He was for a long time a member of the house committee of the London Hospital, and acted as chairman of its Marie Celeste Samaritan Society. Several other hospitals also attracted his active support, among them the Jews' Hospital and Orphan Asylum, on the committee of which he sat at one time.

### IN MEMORY OF FLORENCE NIGHTINGALE.

We are glad to be in a position to announce that a faculty has been granted for the setting up of a marble tablet in East Wellow Church, Hampshire, to the memory of Miss Florence Nightingale. It is to be noticed that the greater the personality which the community wishes to commemorate the more closely, as a rule, does it desire local rather than national memorials. It is not always the best men and women who desire burial in Westminster Abbey.

### A DOCTOR OF ANECDOTES.

The death of Dr. W. Howship Dickinson at the ripe age of eighty removes one who in his time was not only a distinguished London physician, but a very notable personality in St. George's Hospital and its medical school. Possessed of a commanding presence and a due sense of his own dignity, Dr. Dickinson was one of those round whom epigrams and anecdotes seem inevitably to gather. For all his sharp tongue and impatience of stupidity, Dr. Dickinson was a kind-hearted man to whom no trouble in the interests of a patient was ever too great, whether that patient was paying a fee or an inmate of a hospital. His name was best known in connection with Bright's disease and other affections of the kidneys, upon which he was at one time the leading authority; but he was a first-rate all-round physician and a tower of strength to any hospital whose staff he joined. Besides St. George's, he was well known at Great Ormond Street, where he worked right through to the post of senior physician. His only son followed in his footsteps by reaching the assistant staff at St. George's, but died some nine or ten years ago. The latter hospital has also lost another link with the past by the death of Mrs. Caesar Hawkins, widow of one of Queen Victoria's surgeons; Mr. Hawkins was a Fellow of the Royal Society and a really eminent surgeon in the pre-Listerian epoch.

### A NEW DEPARTMENT AT WOLVERHAMPTON HOSPITAL.

The board of management of the Wolverhampton and Staffordshire General Hospital have decided to establish a bacteriological and pathological department to execute work and carry out investigations in these departments of medical science. Such a department, it is felt, will place many facilities within reach of medical men and public authorities who require prompt and accurate reports on specimens they have for examination. A sub-committee has been appointed for making all detailed arrangements and carrying the undertaking into effect at an early date. The department will enable the hospital to fulfil in a high sense the requirements which modern medicine and surgery demand, and, in view of the progress of bacteriology on the one hand, and of the competition which certain firms are instituting by offering to undertake examinations at microscopic fees, should add to the institution's reputation by guaranteeing the quality of the tests and their results. We hope to be in a position to publish full details very shortly.

### NEW SECRETARY OF THE HOUNSLOW HOSPITAL.

Mr. H. J. Baker has been appointed honorary secretary to the Hounslow Hospital in succession to Mr. H. R. Peake. It will be remembered that Mr. Peake sent in his resignation two years ago, but he was induced to continue the work till the completion of the new building, which was expected to have been accomplished at Christmas.

### A NOVEL SUBSTITUTE FOR COVERED WAYS.

An ingenious substitute for a covered way between the administration block and several isolated wards, which is badly needed by the Conway Joint Isolation Hospital Board for their institution in which tuberculosis patients are housed, was suggested by Mr. J. W. Raynes at a meeting last week. "Would it not," he asked, "be better and cheaper to fix up a system of wire ropes with trolleys on which food and other articles could be carried from the kitchen in a covered box?" Mr. Raynes having explained that a similar system was extensively used in the quarries and could be adapted to institutional requirements, Dr. J. R. Williams said that the idea seemed an excellent one. The result is that the engineer, Mr. T. B. Farrington, has been asked to report on the feasibility of the scheme, and many an institution and sanatorium will await his conclusions with interest.

### A COTTAGE HOSPITAL'S ENDOWMENT FUND.

Cottage hospitals often owe very much to individual benefactors, and the latest example is the Felixstowe and Walton Cottage Hospital, which has just received the important addition of £500 to its endowment fund. Attempts to make this fund more adequate have been engaging the efforts of institutional workers in the locality for some time; but it was Mr. C. H. E. Croydon, the donor of the cottage hospital, who succeeded in providing the necessary stimulus. He gave not only the first £50, but promised an additional £50 for every £200 added to the endowment fund. Now that Mr. James Langton, of Carisbrooke, Felixstowe, has received £500 on the hospital's behalf, Mr. Croydon has an opportunity, which no doubt he will embrace, of raising the total to £700, according to his generous promise.

### CHINESE DOCTORS, DRUGS, AND IDEAS.

Some time since we announced that the military authorities in Kwangtung, China, had determined to employ medical students who had either graduated in Europe or America, or had been trained in Western medical science in Hong Kong, and we now learn that the trial has been so satisfactory that no native-trained doctors will be accepted and no native drugs are to be purchased. With regard to the drugs, this will be a sweeping change, seeing that the Chinese *materia medica* is a marvellous thing, consisting of about a thousand various items, classified somewhat as follows:—From minerals about 138 kinds of physic are extracted; from grasses, roots, stubs, leaves, flowers, and seeds, 350 kinds, and so forth. Among the remedies obtained from the animal world some will hardly bear description, but the quainter ones consist of deer's horn pulverised, deer's glue, sheep's milk, hoof of a white horse, thigh of a grey horse, bones, claws, and eyes of the tiger, all converted into nostrums.

### A CELESTIAL AT CAMBRIDGE.

From these and other strange substances the native doctor compounds enormous boluses and draughts, which the unfortunate patient has to swallow. Some of the methods of Chinese native practice are as peculiar as the remedies they employ. The family practitioner is employed by the year, and his fees cease whenever any member of the family by whom he is retained becomes ill. It will be noted that the Celestial doctor is paid by results, and not for restoration to health. His prescriptions are different on different days for the same ailment. This is due to the Chinese belief that the human system varies from day to day. The Chinese doctors contend that there are fifty to sixty kinds of heart disease, twenty or thirty forms of consumption, and at least one hundred varieties of dyspepsia, which is an almost universal complaint in China, due largely to the enormous quantity of tea consumed and to the general practice of eating half-boiled indigestible native cabbage. The adoption of Western medicine and Western-trained doctors by the army and by the officials will soon exercise a potent effect on the population, provided the cost of medicine shows no material increase. The question of economy is always a great factor with the Chinaman, whose wages are small and his ideas of payment in proportion. A further proof of the occidentalisation of China, medically speaking, is the recent appointment of Gnoh Lean Tuck, M.D., B.C., to be director and chief medical officer of the Manchurian Plague Prevention Service. This extremely able and upright official is still remembered at St. Mary's Hospital, where he completed the student period of his medical education; and at Cambridge, where he took a first in honours three years after he arrived in England, hardly able to read and write. It was said at the time that he worked fourteen hours a day all the year round.

### THE EVILS OF PRIVATE PRACTICE.

Under the title of "The Sphere of Institutional Treatment" we begin this week a brief series of articles. Their object is to deal with an apparently hackneyed subject from the unusual point of view of the effect on medical men of certain conditions in private practice. The influence of these on the patient has been very frequently considered, but, though the difficulties of the practitioner have been discussed with a sob or a smile in the privacy of medical circles, the existing evils and their likelihood of remedy have not been dealt with by authority in public. The writer, whose points we must not blunt by quotations in advance, raises the interesting question as to whether the gradual extension of medical treatment in every branch of institutional life holds out any remedy for the evils which he depicts in the current article.

### CASES OF POISONING IN HOSPITALS.

Dr. Firth has analysed the admissions for poisoning, accidental and intentional, into the West London and St. Thomas's Hospitals during recent years; and has constructed an interesting table showing the incidence of 346 cases. Oxalic acid (salt of lemon) heads the list, having been the cause of forty-seven cases; ten of these were accidental and thirty-seven suicidal. This addiction to corrosive acids on the part of would-be poisoners is a curious fact; for the results of drinking oxalic acid are so extremely unpleasant that it might have been thought they would have militated against its popularity. Opium stands next, with forty-one cases; of these thirty-two were deliberate. Hydrochloric acid, another horribly painful corrosive, accounts for thirty-eight cases, thirteen of which proved fatal; indeed this acid proved far more deadly than any of the other drugs used. Liniments of various sorts caused poisoning forty times, nearly always accidentally. Carbolic acid was to blame thirty-three times; only twelve of these were cases of suicide. Ptomaines resulted in twenty-nine admissions, but in not a single fatality. There were many other poisons, but none of them were at all frequently used. Oxalic, hydrochloric, and carbolic acids together form more than one-third of the total, and account for two-thirds of the fatalities among the cases of attempted suicide, from which it would seem obvious that the law dealing with the sale of these deadly drugs needs strengthening. Prussic acid does not appear on the list; doubtless because those who take enough to do themselves harm die so quickly that there is no object in taking them to a hospital.

### DEATH AND PERSONALITY.

If nothing new concerning death has been learnt from Dr. Carrell's much-advertised experiments, the object of which was to keep the vital organs alive after their removal from the organism, they emphasise the lessons which science has already taught. To maintain the phenomena of life in the lungs, heart, and digestive organs of a cat for some twelve hours after the removal from the body may not tell us how life can be prolonged under ordinary conditions, which is the question in which the public seems most interested at present, but it does throw an interesting light upon personality. Imagine the experiment to have succeeded for as many weeks as it did hours, and we are as far off as ever from the personality of the cat. Personality is something more than physical life: it is like the force of an assembly, which exists so long as the majority of the members carry on their functions in the same house and under the same conditions. Death is, in legal and scientific phraseology alike, the dissolution or dispersion of that assembly, the members of which, though still alive, lose on dispersion the power which is more than each one of them considered separately. All forms of contact are forms of creation, and the personality of an organism or of an assembly is the force which results from the contact of its members. Two heads are not merely better than one, they are better than two separately considered heads. And Dr. Carrell's prolongation of life in the separated viscera of a cat is comparable to the Rump Parliament to which Colonel Pride's tactics reduced the House of Commons in 1648. The personality of Parliament, like that of the cat, was destroyed, and how far this mysterious and most valued expression of a man may be beyond the power of those who prolong physical life is well known to all who have studied mental disease or watched the break up of a personality in a body still healthy, which is what we mean by senile decay.

### THE FEES OF PUBLIC VACCINATORS.

The Southwark Board of Guardians has received a reply from the Local Government Board with reference to the deductions made from the fees received by district vaccination officers under the Poor Law Officers' Superannuation Act, 1896, as reported in The Hospital on December 28. The Board was asked whether district public vaccinators or institutional medical officers were entitled when the time arrives to claim superannuation allowances under the Act in respect of vaccination fees. The reply states that save in the case of a medical officer of a Poor-Law institution who performs vaccinations as part of his duties, a public vaccinator who is paid fees under a contract with a Board of Guardians cannot, in the Board's opinion, be regarded as an "officer" as defined by Section 19 of the Poor Law Officers' Superannuation Act. On this view, remuneration received by a district medical officer in respect of services as a public vaccinator is not in ordinary circumstances an emolument of the office of district medical officer for the purposes of the Act. As this reply upholds the decision of Mr. Justice Neville that Guardians are not entitled to grant superannuation allowances in respect of vaccinators' fees, the Board were also asked whether under these circumstances there was any objection to the Guardians returning the deductions made from the vaccination fees of the doctors concerned. To this the Local Government Board reply that where deductions have been made for the purposes of superannuation without legal justification, the money could be refunded without any sanction on their part. This letter of the Local Government Board has settled the question.

### MEDICAL OFFICERS AS PART-TIMERS.

The appointment of Dr. Stanley Foster, a practitioner of the town, to the post of Medical Officer of Health for the Borough of Newport, Isle of Wight, has more than local interest. For it is the outcome of a good deal of discussion arising from the attitude of the Local Government Board. The idea of the Board was that if an officer of a neighbouring authority were appointed the work could be co-ordinated and an officer entirely engaged in public work would be created. The arguments against any important post in the public service being the second interest of an individual, for they are very rarely the first when combined with private practice, are obvious enough. Newport, however, is the central town on the island, and it would seem as if it were jealous of its eminence and hurt at the suggestion that its medical officer should not be a townsman. Tradition is probably on the side of the Corporation, and the Board probably will have to wait till passage of time and increase of population make a whole-time officer admittedly necessary for Newport, Isle of Wight.

### A DEPARTURE IN HOSPITAL ADVERTISING.

It would seem at first sight to the layman almost hopeless to advertise for a gift of the rarest of commodities. Yet there appeared this week a special hospital asking in an advertisement for a gift of radium. There is a high probability that the request will be successful, just as the success of the voluntary hospital system has been largely maintained by inducing the public to take an intelligent interest in institutional affairs. The first hospital to advertise for radium is acting on this principle, and it will be interesting to see if the advertisement be followed by an announcement stating in the well-worn formula that the secretary gratefully acknowledges the receipt of such and such sum for the purchase of radium! The human mind is so constituted that it may easily respond to a plea for radium by a cheque, while remaining unmoved by appeals for gifts of money. All the art of advertising is contained in the recognition of facts like these.

### HOSPITAL MANAGEMENT AT CHELTENHAM.

From time to time we have commented upon the affairs of the Cheltenham General Hospital, and on the sporadic efforts which are being made to reconstruct its management. Last week the question of the position of vice-presidents was again considered, and their right under the present rules to sit on the governing body. Legal opinion has been taken, and its upshot is that vice-presidents can be left off the governing body only upon an alteration of the rules, which however subscribers, upon due notice of the proposed alteration being given, are competent to make. The governors have decided to allow such existing vice-presidents as care to retain their seats to be allowed to do so. The new scheme of management seems to be getting slowly, but, let us hope, surely, into active being.

### LECTURES AT THE CANCER HOSPITAL.

One after another the special hospitals of the Metropolis are starting post-graduate courses of lectures. The latest recruit to the fashion is the Cancer Hospital, Fulham Road, whose staff are to give a lecture apiece during the next ten weeks on various aspects of the treatment of malignant disease. Within the last decade the staff of this hospital has undergone sweeping changes; and there are, in fact, very few of the general teaching hospitals which can produce such an array of surgical talent as the institution in the Fulham Road now can. It is no secret that up to the end of the nineteenth century neither the medical profession nor the patients had very much faith in the personnel of the staff; and it is a proof of the inherent soundness of the voluntary system that so profound a change has been brought about without any interference or pressure from outside sources. The lectures now announced embrace all the common kinds of malignant disease, and promise to be of a thoroughly practical and useful nature. They are free to all practitioners and students of medicine, and will be delivered on Wednesdays at 5 p.m.

### PATIENTS' GIFT TO A SUPERINTENDENT.

During the Christmas festivities at Westmorland Consumption Sanatorium, Grange-over-Sands, the patients presented the medical superintendent, Dr. C. F. Walker, and his wife with a silver rose bowl, suitably inscribed, as a mark of their appreciation of Dr. and Mrs. Walker's efforts to make Christmas a happy time for all. A few days later, at a whist drive for patients and staff, the patients at the home presented the assistant medical officer, Dr. Fitzgerald, with a gold-mounted fountain pen as a permanent reminder of the regard they have for him. Apart from the testimony to the good feeling existing between doctors and patients, which these presentations afford, it is satisfactory to note this practical expression of the pleasure that patients take in the Christmas festivities, which are the object of so much thoughtfulness on the part of institutional workers.

### OBJECT-LESSON IN INSTITUTIONAL FESTIVITIES.

We believe that even our readers, who have been led to expect infinity of resource and the success of high ambitions in institutional entertainments from the accounts already published in our columns, will feel that they have still something to learn when they read an article entitled "The Arts of the Theatre at Fulham Infirmary" on another page. Nor is this effect of surprise and wonder due solely to charm of description. Style apart, few who have ever experimented with amateur dramatic performances will not feel as they read this account that the elaboration connected in the public mind with Sir Herbert Tree's productions on the one hand, and with the many "turns" of the variety theatre on the other, has infected this infirmary's entertainments, so lavish is their scope, so realistic their staging. All the arts of the theatre seem to be represented. If an instance were wanted to show that virtually no feat is impossible to an institution which desires to make its entertainment many-sided and intrinsically good, that instance is found here. If this account of an elaborate performance be studied in connection with Mr. J. H. Smethurst's inspiring article on "The Aim and Working of a Festivities Fund" of two weeks ago, both sides of institutional festivities—the organisation and financing, together with the conduct and performance—are found complete in precept and example.

### CHARGING FEES TO INSURED PATIENTS.

Among the many and, on the whole, unconflicting decisions which have been come to by hospitals as to the treatment of insured persons, the decision which the board of management of the Pembrokeshire and Haverfordwest Infirmary is reported to have arrived at appears as yet to stand alone. Except in urgent cases the decision given is that no insured person will be admitted to the hospital, and that for operations on insured persons a fee of ten guineas is to be charged. The question has been raised whether this fee might not be paid by the Insurance Committee, but the clerk to the Committee is understood to have stated that it could not.

### THIS WEEK'S DRUG MARKET.

Few changes in value of any note have taken place during the week, but a fair business has been done; and, judging from the number of inquiries for various drugs, it is not improbable that orders will follow in due course. The demand for quinine continues, and a large business has been done; but makers have announced no advance in price. Essence of lemon is again dearer. The Opium market has been quiet, with a tendency towards lower prices; a fair business has been done in morphine. Sugar of milk tends lower in price.

### TO OUR READERS.

Contributions are specially invited from any of our readers to these columns. They should deal with topical subjects and news. They must be authenticated for the information of the Editor only. The minimum payment if published is 5s. There is no hard-and-fast-rule as to space, but notes of about twenty lines in length are preferred.

